# Effect of ciprofol compared with propofol on hemodynamics in bronchoscope procedures during anesthetic induction: a randomized double-blind controlled study

**DOI:** 10.3389/fmed.2025.1545736

**Published:** 2025-05-16

**Authors:** Fang Chen, Fang Xing, Jiayu Huang, Junwen Zhu, Ye Jiang, Cheng Li

**Affiliations:** Shanghai Key Laboratory of Anesthesiology and Brain Functional Modulation, Department of Anesthesiology and Perioperative medicine, Clinical Research Center for Anesthesiology and Perioperative Medicine, Shanghai Fourth People’s Hospital, School of Medicine, Translational Research Institute of Brain and Brain-Like Intelligence, Tongji University, Shanghai, China

**Keywords:** bronchoscope, ciprofol, general anesthesia, propofol, hemodynamics

## Abstract

**Objective:**

Propofol poses hemodynamic challenges and injection pain during anesthesia. This study compared the effects of propofol and ciprofol, a novel propofol analog, on hemodynamics in painless bronchoscopy during induction of general anesthesia.

**Methods:**

A total of 250 patients underwent painless bronchoscopy anesthesia from October 2021 to June 2023. Randomly assigned to ciprofol or propofol groups, they received respective anesthesia. Changes in heart rate, blood pressure pre- and post-induction, after laryngeal mask airway placement, before and after fiberoptic scope insertion, incidence of choking and injection pain, vasoactive drug use, and satisfaction levels of operators, anesthesiologists, and patients were compared.

**Results:**

Ciprofol group showed significantly higher blood pressure 3 min post-induction compared to propofol group (80.81 ± 12.49 mmHg vs. 84.47 ± 12.80 mmHg, *p* = 0.023), with no significant difference post-bronchoscope placement. Injection pain incidence was significantly lower with ciprofol (0.8% vs. 37.1%, *p* < 0.001). Operators, anesthesiologists, and patients in ciprofol group reported higher satisfaction.

**Conclusion:**

Ciprofol exhibits less hemodynamic impact and injection pain than propofol, suggesting it as a viable alternative for anesthesia induction in bronchoscopy under general anesthesia with a laryngeal mask airway.

**Clinical trial registration:**

https://clinicaltrials.gov/, identifier [ChiCTR2200063048].

## 1 Introduction

The importance of fiberoptic bronchoscopy in diagnosing and treating respiratory diseases has steadily risen. With increasing patient demand and the complexity of diagnostic and therapeutic procedures, there’s a growing need for painless fiberoptic bronchoscopy (1–3). Currently, fiberoptic bronchoscopy under general anesthesia is well-established. Patients requiring this examination are often old with reduced multisystem function. Hence, the optimal anesthesia for painless fiberoptic bronchoscopy aims to swiftly attain deep anesthesia while ensuring hemodynamic stability, rapid awakening, and minimal residual drug effects.

Propofol, a conventional intravenous general anesthetic, is widely used in outpatient and inpatient settings, including painless procedures such as abortion, gastrointestinal endoscopy, and fiberoptic bronchoscopy (4, 5). Propofol typically has an onset time of 0.5–1 min and a maintenance duration of 5–10 min. It undergoes rapid distribution with a half-life period of 2–4 min and rapidly eliminates with a half-life period of 30–60 min (6, 7). Propofol’s notable circulatory inhibition often leads to a significant drop in blood pressure, even severe hypotension, especially in elderly patients (1, 8–13). This complication may lead to multi-organ dysfunction, unless treated with vasoactive and/or inotropic drugs. Additionally, injection pain with propofol affects a substantial proportion of adults, with incidence rates ranging from 28 to 90% (14–17).

With the increasing demand for painless endoscopy, driven by factors such as aging populations and rising comorbidities, anesthesiologists require sedative drugs with precise efficacy, mild respiratory depression, stable circulation, minimal injection pain, and high-quality awakening.

Ciprofol (HSK3486) is a novel 2,6-bisubstituted phenol derivative used for anesthesia, developed by Haisco Pharmaceutical Group Co. Ltd., Chengdu, China. Acting as a structural analog of propofol, it functions as a γ-Aminobutyric acid (GABA) receptor potentiator. By enhancing GABA receptor-mediated ion channels, it facilitates chloride influx, inducing hyperpolarization of nerve cell membranes and central neural inhibition (18). *In vitro* studies indicate that ciprofol’s binding capacity to GABA-A receptors is higher than that of propofol, and its sedative/anesthetic potency is 4–5 times greater than propofol (19–21). Within 2 min of administration, the sedation scores rapidly decreased to a minimum (0 or 1), gradually recovering thereafter. Half of the subjects achieved a MOAA/S (Modified Observer’s Assessment of Alertness/Sedation) score ≤ 1 at 6 min post-administration, returning to 5 (fully awake) at 10 min. All subjects reached an MOAA/S score of 5 at 14 min post-administration (22, 23). Multiple phase I–III clinical trials conducted in China and Australia have demonstrated the following (19, 24, 25):

(1) It exhibits a rapid onset of action, with a comparable successful induction time to propofol (1.09 min vs. 1.13 min) and approximately 3 min of awakening time. The 0.4–0.9 mg/kg ciprofol administration regimen was well-tolerated and exhibited rapid working and recovery properties.
(2) Its potency is 4–5 times greater than propofol, with effects equivalent to 1.5–2 mg/kg of propofol at doses of 0.4–0.5 mg/kg, resulting in a significant reduction in narcotic drug use.
(3) There is a lower incidence of adverse events related to respiratory depression compared to that by propofol (1.6% vs. 4.6%).
(4) Ciprofol demonstrates less injection pain, occurring in only 1/10th of cases compared to that by propofol (4.9% vs. 52.4%). It achieves rapid anesthesia onset, smooth awakening, a low incidence of injection pain (20, 23, 26, 27) and related with several adverse events, such as hypotension, abnormal body movements, sinus bradycardia, as well as prolonged QTc interval (25). These results from various studies suggest that ciprofol exhibits favorable anesthetic characteristics, efficacy, and safety.

However, whether ciprofol’s effect on hemodynamics of patients during anesthesia induction undergoing bronchoscope procedures is superior to that of propofol remains unclear. Our study aims to investigate and compare the impact of propofol and ciprofol with equivalent doses on hemodynamics in patients undergoing painless fiberoptic diagnosis and treatment.

## 2 Materials and methods

### 2.1 Patients

A total of 250 patients who underwent elective bronchoscopy procedures with laryngeal mask anesthesia airway in our hospital from September 2022 to June 2023 participated. Before patient’s enrollment, this study was approved by the hospital’s ethics committee (2021108-001) and was registered in the Chinese Clinical Trials Registry (ChiCTR2200063048, Fang Chen, 2022/08/29). All patients signed written informed consent.

### 2.2 Inclusion and exclusion criteria

#### Inclusion criteria

(1) Those patients who accepting selective diagnostic and/or therapeutic bronchoscopy with laryngeal mask anesthesia;
(2) Patients who met the criteria for American Society of Anesthesiologists grade I–III, aged from 18 to 80 years;
(3) Body mass index (BMI) from 18 to 30 kg/m^2^;
(4) Respiratory rate between 10 and 24 breaths/min during screening and baseline periods; SpO_2_ ≥ 93% while breathing air; Systolic blood pressure (SBP) ≥ 90 mmHg; Diastolic blood pressure (DBP) ≥ 55 mmHg; Heart rate between 50 and 100 bpm;
(5) Willing to sign the informed consent, and following the study until completion.

#### Exclusion criteria

(1) Had contraindications or history of accidents to deep sedation/general anesthesia;
(2) Known allergies to eggs, soy products, opioids and their rescue medications, as well as propofol; Propofol, opioids, and their rescue medications for patients with contraindications;
(3) Patients who had been intubated and/or mechanically ventilated before bronchoscopy;

Patients with one or more increased sedation/anesthesia risks in the pre-/baseline period were screened for the following situations:

(a) Cardiovascular disease history: uncontrolled hypertension (defined as SBP ≥ 170 mmHg and/or DBP ≥ 105 mmHg without antihypertensives, or SBP > 160 mmHg and/or DBP > 100 mmHg after antihypertensives), New York Heart Association (NYHA) cardiac function grade ≥ III, severe arrhythmia, heart failure, acute myocardial ischemia, unstable angina, myocardial infarction within nearly 6 months before screening, history of tachycardia/bradycardia requiring medical therapy, high-grade atrioventricular block, or QTc interval ≥ 450 ms, etc.;
(b) Respiratory disease history: severe chronic obstructive pulmonary diseases well as acute exacerbation, severe airway stenosis, pharyngolaryngeal lump, severe respiratory infection within 2 weeks before screening, etc.;
(c) History of neurological and psychiatric diseases: head injury, convulsion, intracranial hypertension, cerebral aneurysm, and history of cerebrovascular accident, schizophrenia, mania, long-term use of psychotropic drugs, cognitive dysfunction, etc.;
(d) Gastrointestinal disease history: presence of gastrointestinal retention, active bleeding, history of gastroesophageal reflux or obstruction that could result in countercurrent mistake inhalation;
(e) Uncontrolled disease histories of significant clinical significance involving the liver, kidneys, hematologic, nervous, or metabolic systems that, in the judgment of the investigator, may not be appropriate for study participation;
(f) History of excessive drinking, over 2 units of alcohol each day (1 unit = 360 mL of beer or 45 mL of liquor or 150 mL of wine with > 40% alcohol) within 3 months before the screening stage;
(g) Drug abusing within 3 months before the screening stage;
(h) Experience of blood transfusion within 2 weeks before the screening stage;

(5) The screening/baseline stage was at risk for the following respiratory management:

(a) asthma exacerbations;
(b) Those with sleep apnea syndrome;
(c) Had a history or family history of malignant hyperthermia;
(d) Had experience with endotracheal intubation failure;
(e) Presence of difficult airway as judged by the investigator (e.g., modified Mallampati score grade ≥ III);

(6) Screening period/baseline period used any of the following medications or treatments:

(a) Involved in any other medication clinical investigation within 1 month prior to screening;
(b) Those who had used propofol, other sedatives, and/ or opioids or compounded formulations containing analgesic components within 72 h prior to baseline;

(7) Screening period/baseline period laboratory test indicators met the following criteria and reconfirmations:

(a) white blood cell count ≤ 3.0 × 10^9^/L;
(b) Platelet count ≤ 80 × 10^9^/L;
(c) Hemoglobin ≤ 80 g/L;
(d) Prothrombin time ≥ 1.5 × upper limit of normal (ULN);
(e) Activated partial thromboplastin time ≥ 1.5 × ULN;
(f) Alanine transaminase and/or aspartate transaminase ≥ 3 × ULN;
(g) Total bilirubin ≥ 1.5 × ULN;
(h) And serum creatinine ≥ 1.5 × ULN;
(i) Pregnant and nursing mothers; Those who of childbearing potential were unwilling to contraception; Or patients with a pregnancy plan within 3 months of the termination of the study (containing male patients);

(8) Those who have any other factor that would not be appropriate for participation in this clinical study in the opinion of the investigator.

### 2.3 Sample size calculation

The incidence of hypotension using propofol for general anesthesia and sedation is 36–43.3% (average 39.65%) (28, 29). Assuming an α-value of 0.05 with a statistical power of 90% and a relative risk of 0.5 or lower for decreasing in the incidence of hypotension in ciprofol group vs. propofol group. Considering a potential dropout rate due to possible adverse events and serious adverse events at around 10%, we estimated that each group should have a sample size close to 121 participants. To obtain more accurate experimental results, an integer value of 125 was chosen for each group simultaneously. Thus, altogether 250 patients will be enrolled in this study. Methods of this study have been published (30).

### 2.4 Randomization and blinding

Participants will be assigned to the propofol group or ciprofol group (1:1 ratio) using a random number table generated by computer through the allocation manager, who will store the randomization table. Patients will be informed preoperatively that they will be randomly assigned to either group. The randomization numbers will only be provided to a specific nurse who will prepare the study medicine in a closed room until the end of the trial and will not be involved in data analysis. Patients, anesthesiologists, surgeons, data collectors, independent statisticians, and evaluators were independent from patient allocation. The grouping information was known only by the allocation manager and the specific nurse. Independent statisticians will solely focus on medicine efficacy without knowing any allocation information. The evaluators would not have access to unblock the blinding.

### 2.5 Study protocol

**FIGURE 1:**
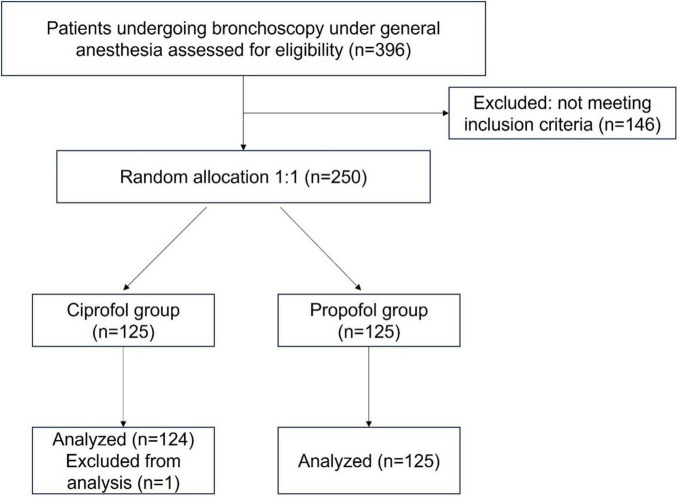
Trial enrollment flow diagram.

Patients were informed preoperatively of their random assignment to either group. Randomization numbers were provided only to a specific nurse responsible for preparing the study medicine in a closed room until the end of the trial; this nurse was not involved in data analysis. The whole study flow chart is present in Figure 1.

In the ciprofol group, the test drug was diluted to 40 mg/20 mL with normal saline by the investigator. During the induction period, patients received a slow bolus intravenous injection within 30 s at a dose of 0.2 mL/kg (0.4 mg/kg).

In the propofol group, propofol was administered as a slow bolus within 30 s at the induction dose of 0.2 mL/kg (2 mg/kg) by intravenous injection. All patients received routine premedication and peripheral veins were cannulated before anesthesia induction. Electrocardiogram, pulse oximetry, as well as noninvasive blood pressure were monitored routinely, with a monitoring interval of 1 min. Anesthesia induction involved sequential intravenous bolus administration of ciprofol or propofol (0.2 mL/kg), sufentanil (0.2 μg/kg), and cisatracurium (0.05 mg/kg). After 3 min, a laryngeal mask airway was placed. Intermittent positive pressure ventilation was initiated, with breathing parameters set to a tidal volume of 6 mL/kg, a frequency of 12 bpm, and an oxygen flow of 2.0 L/min with 100% oxygen concentration.

Additional experimental medication (0.1 mL/kg) was administered 1 min before the fiberoptic scope entered the glottis through the laryngeal mask airway, consisting of propofol (4–12 mg/kg⋅h) and remifentanil (0.05–2 μg/kg⋅min) for maintenance of anesthesia. Postoperative antagonists, including neostigmine (0.03 mg/kg), atropine (0.015 mg/kg), and nalmefene (0.5–1 μg/kg), were given routinely. The patient was considered awake when spontaneous breathing returned and could follow instructions to complete movements. The laryngeal mask airway was then removed. Ephedrine (6–12 mg) or phenylephrine (20–100 μg) boluses were administered if blood pressure fell below 20% of the baseline value and could be repeated as needed. Additional test medication (2–4 mL) could be given singly if blood pressure exceeded 1/5 of the basic value. When the heart rate fell below 50 bpm, atropine (0.3–0.5 mg) was administered.

### 2.6 Data collection

(1) Recording mean arterial pressure (MAP) and heart rate (HR) at following time points: before anesthesia induction (T0), 1 min (T1), 2 min (T2), and 3 min (T3) after injection of induction drugs, immediately after laryngeal mask placement (T4), immediately before bronchoscope placement (T5), and immediately after bronchoscope placement (T6).
(2) Incidents of patient choking during laryngeal mask and fiberoptic scope placement, injection pain, and vasoactive drug usage were documented.
(3) The satisfaction levels of the clinic operator, anesthesiologist, and patient were recorded.

### 2.7 Outcomes

#### 2.7.1 Primary outcome

The MAP and HR in all patients at various time points during the anesthesia induction until bronchoscope placement.

#### 2.7.2 Secondary outcomes

Secondary outcomes included incidents of patient choking during laryngeal mask and fiberoptic scope placement, injection pain, and vasoactive drug usage in all patients. The satisfaction levels of the clinic operator, anesthesiologist, and patient were also recorded as secondary outcomes.

### 2.8 Statistics

Statistical analysis was performed by SPSS version 25.0 (IBM Corporation, Armonk, NY, USA). Measurement data were expressed as mean ± standard deviation, and independent samples *t*-tests were performed to compare two groups. Counting data were expressed as absolute values, and comparisons between groups were analyzed using the chi-square test. Statistical significance was defined as *P* < 0.05. The *post-hoc* subgroups included age and ASA class.

## 3 Results

### 3.1 Comparison of general characteristics

With one excluded because of the low hemoglobin, 249 patients were intake in this study and randomized into two groups: the ciprofol group (*N* = 124) and the propofol group (*N* = 125) eventually. All of patients finished the procedures (shown in Figure 1). No statistic differences existed in gender ratio, age, BMI, hemoglobin concentration, basal HR and MAP between two groups (Table 1). Biomarks of liver and kidney in two groups were in normal range, before or after bronchoscope procedures shown in Table 1.

**TABLE 1:** Baseline characteristics of patients.

Variables	Propofol group (*n* = 124)	Cipropol group (*n* = 125)	*P*
Male/female	71/53	73/52	0.898
Age (year)	62.90 ± 11.79	62.70 ± 12.77	0.894
BMI (kg/m^2^)	23.01 ± 3.51	22.24 ± 5.31	0.177
HB (g/mL)	128.52 ± 17.26	126.61 ± 16.87	0.377
HR (T0, bpm)	78.56 ± 13.75	79.59 ± 14.25	0.563
MAP (T0, mmHg)	98.97 ± 12.70	99.86 ± 12.66	0.580
**Before bronchoscope procedures**			
ALT (U/L)	24.90 ± 18.02	24.92 ± 8.74	0.990
AST (U/L)	24.67 ± 10.11	22.98 ± 6.39	0.115
Cr (μmol/L)	65.97 ± 17.81	65.21 ± 21.73	0.762
Bun (mmol/L)	5.56 ± 1.84	5.27 ± 1.84	0.212
**After bronchoscope procedures**			
ALT (U/L)	25.17 ± 18.35	25.41 ± 10.79	0.902
AST (U/L)	26.38 ± 10.51	24.33 ± 6.91	0.07
Cr (μmol/L)	65.64 ± 16.66	63.39 ± 19.43	0.326
BUN (mmol/L)	5.50 ± 1.78	5.42 ± 5.62	0.878

Data are expressed as means ± standard deviation. BMI, body mass index; HB, Hemoglobin; HR, heart rate; MAP, mean arterial pressure; ALT, alanine aminotransferase; AST, aspartate transaminase; Cr, creatinine; BUN, blood urea nitrogen.

### 3.2 Primary outcome

**FIGURE 2:**
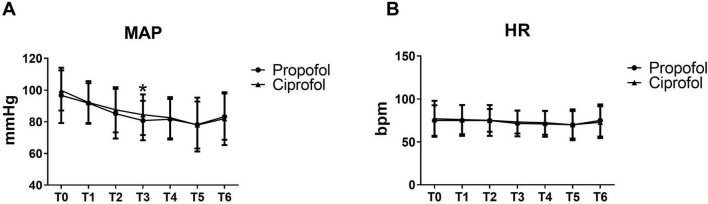
MAP was lower in the propofol group than ciprofol group at T3. **(A)** Mean arterial pressure at different times in two groups. *P < 0.05 vs. propofol group. **(B)** Heart rate at different times in the propofol group and ciprofol group.

The MAP at 3 min after administration (T3) was significantly lower in the propofol group than ciprofol group (80.81 ± 12.49 mmHg vs. 84.47 ± 12.80 mmHg, *P* = 0.023) in Figure 2. No statistically significant differences were observed between two groups in HR and MAP before induction (T0), 1 min after administration (T1), 2 min after administration (T2), 4 min after administration (T4), or 5 min after administration (T5).

### 3.3 Secondary outcomes

Incidence of injection pain was significantly lower in the ciprofol group, while higher in the propofol group (0.8% vs. 37.1%, *P* < 0.001) in Table 2. Between two groups, no significant differences were observed in the incidence of choking was not significantly different (32.3% vs. 29.6%, *P* = 0.682), and the use of vasoactive drugs (33.1% vs. 22.4%, *P* = 0.067).

**TABLE 2:** Incidence of choking, injection pain, and vasoactive drug use in both groups.

Adverse events	Propofol group (*n* = 124)	Ciprofol group (*n* = 125)	*P*
Chocking (*n*, %)	40 (32.3%)	37 (29.6%)	0.682
Injection pain (*n*, %)	46 (37.1%)*	1 (0.8%)	<0.001
Vasoactive drug using (*n*, %)	41 (33.1%)	28 (22.4%)	0.067

Data expressed as number of patients (%).

*Indicates *p* < 0.05 between the two groups.

### 3.4 Satisfaction of operator, anesthesiologist, and patient between two groups

Overall, 38.4% of patients receiving ciprofol were satisfied compared with only 0.8% in the propofol group (*P* < 0.001). Similarly, 31.2% of operating surgeons and 33.6% of anesthesiologists in the ciprofol group were satisfied, compared with 0.8% in the propofol group (*P* < 0.001 for both comparisons). Thus, the ciprofol group showed significantly higher satisfaction levels among operators, anesthesiologists, and patients. Most patients, operators, and anesthesiologists in both groups were satisfied, with a few patients showing general satisfaction, and no cases of dissatisfaction occurred in either group (Table 3).

**TABLE 3:** Satisfaction of patients, clinic operators, and anesthesiologists in the propofol and ciprofol groups.

	(*n*, %)	Propofol group	Ciprofol group
Patient	Very satisfactory	1 (0.8%)	48 (38.4%)
	Satisfactory	119 (96.0%)	76 (60.8%)
	Common	4 (3.2%)	1 (0.8%)
	Unsatisfactory	0 (0%)	0 (0%)
Clinic operator	Very satisfactory	1 (0.8%)	39 (31.2%)
	Satisfactory	105 (84.7%)	78 (62.4%)
	Common	18 (14.5%)	8 (6.4%)
	Unsatisfactory	0 (0%)	0 (0%)
Anesthesiologist	Very satisfactory	1 (0.8%)	42 (33.6%)
	Satisfactory	123 (99.2%)	81 (64.8%)
	Common	0 (0%)	3 (2.4%)
	Unsatisfactory	0 (0%)	0 (0%)

Data are expressed as number of patients, operators, and anesthesiologists (%).

### 3.5 Subgroup analysis

Table 4 showed the subgroup analysis of injection pain incidence. The results showed no significant subgroup interaction effects between age and ASA class, *p-*values for interaction were 0.580 and 0.604, respectively. Subgroup interaction analysis of MAP at T3 was showed in Table 5. Stratify results by age showed MAP at T3 was significantly higher in the ciprofol group than in the propofol group, in aged over 60 patients while not in populations with age ≤ 60. Among populations with ASA class II, MAP was significantly higher in the ciprofol group than in the propofol group, while not in populations with ASA class III. *P*-values for interaction were 0.679 and 0.552, respectively.

**TABLE 4:** Subgroup analysis for incidence of injection pain.

Subgroups	Ciprofol group		Propofol group		*P*	*P* for interaction
	**No**	**Case (%)**	**No**	**Case (%)**		
Age (years)						0.580
<60	34	0 (0)	36	12 (33.33)	<0.001	
≥60	91	1 (1.10)	88	34 (38.64)	<0.001	
ASA						0.604
2	102	0 (0)	102	37 (36.27)	<0.001	
3	23	1 (4.35)	22	9 (40.90)	0.001	

Data expressed as number of patients (%).

**TABLE 5:** Subgroup analysis for mean arterial pressure at T3.

Subgroups	Ciprofol group		Propofol group		*P*	*P* for interaction
	**No**	**Mean ± SD (mmHg)**	**No**	**Mean ± SD (mmHg)**		
Age (years)						0.679
<60	34	84.96 ± 10.78	36	82.14 ± 13.31	0.335	
≥60	91	84.29 ± 13.53	88	80.27 ± 12.17	0.038	
ASA						0.552
2	102	84.86 ± 12.00	102	80.75 ± 12.04	0.015	
3	23	82.78 ± 16.05	22	81.11 ± 14.71	0.717	

BMI, body mass index; DBP, diastolic blood pressure; GABA, γ-Aminobutyric acid; HR, heart rate; LMA, laryngeal mask airway; MAP, mean arterial pressure; MOAA/S, Modified Observer’s Assessment of Alertness/Sedation; SBP, systolic blood pressure; ULN, upper limit of normal.

## 4 Discussion

Our study compared ciprofol and propofol regarding changes in blood pressure, HR, choking, injection pain, vasoactive drug use, and patient, doctor, and anesthesiologist satisfaction during the induction of anesthesia with elective laryngeal mask in fiberoptic bronchoscopy.

In this study, patients exhibited significantly lower MAP at 3 min after administration (T3) in the ciprofol group than propofol group. However, there were no statistic differences in MAP between two groups from the fourth minute post-administration. Both groups experienced a reduction in blood pressure to a minimum at the fifth minute post-administration, consistent with drug metabolism. Blood pressure then increased at the sixth minute, coinciding with bronchoscope placement, indicating operant stimulation. In subgroup analysis, stratify results by age and ASA class showed different significance. In patients aged over 60 and with ASA class II, ciprofol showed benefits for blood pressure. We did not take into account for subgroup analysis in study design stage, therefore interaction effects need further investigation in future.

This result suggests that ciprofol caused a slower decline in blood pressure, but the final decline was consistent with propofol. However, the usage of vasoactive drugs showed no difference statistically between two groups. The circulatory inhibition by ciprofol was resemble that by propofol, with a comparable onset time, and the blood pressure decreased slightly more slowly than that by propofol. Hemodynamic instability caused by propofol during the induction have been reported. Previous studies also showed circulatory stability of ciprofol compared with propofol during anesthesia induction in selective surgeries (24, 31, 32).

In other painless endoscopic studies, it was observed that the incidence of blood pressure drop due to ciprofol was dose-dependent. The incidence of hypotension in the 0.4 mg/kg ciprofol dose group was significantly higher than other two smaller dose groups (22). A previous study suggested a dose of 0.3 mg/kg ciprofol for older patients, which demonstrated comparable efficacy to the 0.4 mg/kg dose administered to younger patients (33). Given that the average age of patients in this study was 62, the ciprofol dose for the older population should be appropriately reduced. This adjustment may promote hemodynamic stability during the induction period. In a clinical study, ciprofol was administered at 0.1–0.4 mg/kg⋅h in the maintenance period, demonstrating a comparable intraoperative hemodynamic profile to propofol (23). Therefore, further studies should be investigated in the influence of ciprofol during anesthesia maintenance in patients undergoing bronchoscopy.

In a recent phase III noninferiority trial, the incidence of injection site pain was lower in patients receiving ciprofol than in patients receiving propofol (18.0% vs. 77.1%), during anesthesia induction (34). In our study, incidence of injection pain was obviously lower in the ciprofol group than propofol group (0.8% vs. 37.1%, *P* < 0.001), consistent with previous findings in painless endoscopy studies (24). Subgroup analysis results showed no interaction effects of age and ASA class. Many factors may result in injection pain of propofol, such as the injection site, dimension of blood vessel, injection speed, concentration of propofol in the blood (14). Previous study suggested that intravenous injection of lidocaine could mitigate propofol-related injection pain (35–37), but may reduce patients’ satisfaction because of metallic taste (38). Ciprofol binds to GABA_*A*_ receptors more tightly than propofol does and exhibits reduced lipophilicity and a more suitable steric bulk (39). Due to the high hydrophobicity and low plasma concentration of ciprofol, it may reduce the occurrence of injection pain (19, 20, 40). Injection pain can result in adverse effect like tension and body movements, with the potential to impact hemodynamic stability during induction (41). It may be a possible cause for ciprofol slight hemodynamics fluctuation. Other adverse events such as hypoxemia, bradycardia, abnormal body movement and so on, have been reported previously (33, 42), which were not recorded in this study.

Owing to the brief duration of bronchoscopy in our study, a low dose of cisatracurium (0.05 mg/kg) was administered during the induction period, which may not have adequately suppressed the patients’ choking reflex during fiber optic placement. Consequently, choking occurred upon fiberoptic placement in both groups, with no significant difference observed between them (32.3% vs. 29.6%, *P* = 0.682). Regarding satisfaction, the majority of patients, operators, and anesthesiologists in both groups expressed satisfaction. Propofol could bring euphoric moods to patients, which can improve comfort (43–45). However, patients, operators, and anesthesiologists in the ciprofol group were more satisfied than those in the propofol group. One randomized controlled trial in patients receiving fiberoptic bronchoscopy also displayed that patients were more satisfied in the ciprofol group than propofol group (46). This finding aligns with the less injection pain of ciprofol, suggesting that reducing injection pain can substantially enhance patient satisfaction. Less injection site pain of ciprofol could lead to high satisfaction of patient because most patients can recall injection pain after waking up in previous studies (47–49). Moreover, besides improving patient comfort and compliance, the notable reduction in injection pain has minimized the need for additional interventions to manage or prevent injection pain, streamlining procedures and enhancing the experience for both operators and anesthesiologists (40).

This study also has a little of limitations. Firstly, being a single-center clinical study, its results may lack generalizability, and a multi-center approach would enhance the breadth of the findings. Secondly, this study only focused on specific adverse events such as injection pain, choking reactions, and MAP reduction requiring vasoactive drugs, neglecting other potential adverse reactions like elevated blood bilirubin, prolonged QT interval, muscle twitching, and dizziness. Thirdly, we just paid attention to induction not full periods of general anesthesia. The effect of ciprofol during maintenance of anesthesia in patents received bronchoscope procedures needed further study, Additionally, while the inclusion criteria allowed for patients aged 18–80 years, 72.8% of patients in the ciprofol group aged over 60 years and 71.8% in the propofol group aged over 60 years. The majority of participants were older adults, with the average age being 62.7 years in the ciprofol group and 62.9 years in the propofol group. This skew toward older patients might limit the generalizability of the study’s findings to younger populations. At last, combination of drugs has been investigated in several studies (50–52). The combination effect of ciprofol and other anesthetics in fiberoptic bronchoscopy procedures should be focused on in future. Therefore, further research is needed to investigate the impact of ciprofol across different age groups and populations to elucidate its safety profile in fiberoptic bronchoscopy diagnosis and treatment.

## 5 Conclusion

In summary, ciprofol exhibited a less pronounced inhibitory effect on patients’ circulatory systems and demonstrated significant superiority over propofol in reducing injection pain during induction of general anesthesia. Therefore, it can be safely utilized for anesthesia induction in patients undergoing elective fiberoptic bronchoscopy under general anesthesia with laryngeal mask airway.

## Data Availability

The raw data supporting the conclusions of this article will be made available by the authors, without undue reservation.
